# Morphometric characteristics of the thoracοlumbar and lumbar vertebrae in the Greek population: a computed tomography-based study on 900 vertebrae—“Hellenic Spine Society (HSS) 2017 Award Winner”

**DOI:** 10.1186/s13013-019-0176-4

**Published:** 2019-02-19

**Authors:** Theodoros B. Grivas, Olga Savvidou, Stefanos Binos, Georgios Vynichakis, Dimitrios Lykouris, Michail Skaliotis, Eleni Velissariou, Konstandinos Giotopoulos, Konstandinos Velissarios

**Affiliations:** 1 Orthopaedics and Traumatology Department, “Tzaneio” General Hospital of Piraeus, Piraeus, Greece; 2 National and Kapodistrian University of Athens, First Department of Orthopaedics, “Attikon” University Hospital, Rimini 1, Chaidari, Athens Greece; 3 Innovative Medical Technologies, Holargos, Athens Greece; 4 Queen Mary University of London, London, UK

**Keywords:** Morphometric characteristics, Thoracolumbar vertebrae, Lumbar vertebrae, Greek population, A computed tomography-based study

## Abstract

**Background:**

Vertebrae morphology appears to have genetic and ethnic variations. Knowledge of the vertebra and pedicle morphology is essential for proper selection and safe application of transpedicular screws. The aim of this study is to create a morphometric database for thoracolumbar and lumbar vertebrae (T9–L5) among individuals of both sexes in the Greek population.

**Material and methods:**

The morphometric dimensions of T9–L5 vertebrae on computed tomography (CT) scan images were measured in 100 adults (79 males and 21 females), without spinal pathology, age from 33 to 87 years old (mean 70 ± 8.73 years). The anterior vertebral body height (AVBH), the posterior vertebral body height (PVBH), the angle formed by the upper end plate of vertebral body and the horizontal line in the sagittal plane, the inner cancellous and outer cortical pedicle height and width, the angle formed by the longitudinal trajectory of the right- and left-sided pedicles and the midline anteroposterior axis of the vertebra (pedicle axis angle (PAA)), and the postero-anterior trajectory’s length of the pedicle from the entry point to the anterior cortex of the vertebra (PTLP), for the right- and left-sided pedicles, were calculated. The Mann-Whitney *U* tests were conducted to compare the differences in various morphometric characteristics between sexes. The collected data were statistically analyzed using the SAS/STAT software 3.1.3 and SPSS version 22. The statistical significance was set at the level of *p* < 0.05. The intra- and inter-observer reliability of the measured parameters was also calculated.

**Results:**

The L5 vertebra had the maximum AVBH with a mean of 28.47 mm (SD ± 2.55 mm) in males and 26.48 mm (SD ± 1.61 mm) in females. The maximum *PVBH* in males was at L1 vertebra with a mean of 27.77 mm (SD ± 1.64 mm) and in females at L2 vertebral with a mean of 27.11 mm (SD ± 1.27 mm). Regarding the *left pedicle dimensions*, the maximum *inner cancellous* and *outer cortical pedicle height* was at T11 with a mean of 12.86 mm (SD ± 1.26 mm) and 18.82 mm (SD ± 1.37 mm) in males and 10.24 mm (SD ± 1.88 mm) and 16.19 mm (SD ± 3.27 mm) in females, respectively. The *maximum inner cancellous* and *outer cortical pedicle width* was at L5 with a mean of 11.57 mm (SD ± 1.97 mm) and 17.08 mm (SD ± 1.97 mm) in males and 10.24 mm (SD ± 1.88 mm) and 16.27 mm (SD ± 3.27 mm) in females, respectively. The *largest PAA* was found at the L5 with a mean angle of 26.23° (SD ± 2.65°) in males and 23.63° (SD ± 4.59°) in females, respectively. The *maximum PTLP* was found at the level of L4 with a mean of 55.31 mm (SD ± 4.52 mm) in males and 48.7 mm (SD ± 4.17 mm) in females, respectively. Regarding the *right pedicle dimensions*, the maximum inner cancellous and outer cortical pedicle height was found at T12 with a mean of 13.03 mm (SD ± 2.01 mm) and 18.01 mm (SD ± 1.56 mm) in males and 10.24 mm (SD ± 1.23 mm) and 16.14 mm (SD ± 1.23 mm) in females, respectively. The maximum inner cancellous and outer cortical pedicle width was at L5 with a mean of 11.3 mm (SD ± 2.86 mm) and 16.34 mm (SD ± 2.98 mm) in males and 12 mm (SD ± 3.18 mm) and 15.69 mm (SD ± 2.59 mm) in females, respectively. The *greater PAA* was at the L5 vertebral with a mean of 25.7° (SD ± 5.19°) in males and 25.56° (SD ± 5.31°) in females, respectively. The *maximum PTLP* was at the level of L3 with a mean of 54.86 mm (SD ± 3.18 mm) in males and 49.01 mm (SD ± 2.97 mm) in females, respectively. At all vertebrae, the only statistically significant difference (*p* < 0.0001) between the two sexes was the mean PTLP of the right and the left pedicle. The L5 vertebra was found to have the largest AVBH, PAA, and pedicle width in male and female populations.

**Conclusions:**

This study provides a database of morphometric characteristics on thoracolumbar and lumbar vertebrae from T9 to L5 in the Greek population. This database may prove to be of great significance for forthcoming comparative studies. It can also serve as a basis in order to detect pathological changes in the spine and furthermore to plan operative interventions. It was found that the dimensions of thoracolumbar and lumbar vertebrae in the Greek population are sex-dependent. In the current study, vertebra and pedicle dimensions seem to have some similarities compared to other Western populations. However, in the thoracolumbar region, the pedicles of T9 and T10 may hardly accommodate a 4.00-mm pedicle screw given the narrow inner cancellous pedicle width. Importantly, the vertebra and pedicle dimensions measured in the current study can be used to guide the selection of transpedicular screws in the Greek population and to guide further research.

## Introduction

Transpedicular screws are commonly used for posterior fixation in spinal fractures, deformity, instability, and degenerative disease [1–4]. However, their insertion is demanding, with the reported malpositioning rate up to 11%, based on postoperative CT assessment [5, 6] and the overall incidence of neurological, visceral, and vascular complications up to 42% [2, 7]. Genetic and ethnic variations in width, height, and orientation of vertebra and pedicle dimensions have been documented [1, 2, 4, 5]. The morphometric analysis of the vertebrae and pedicles is a clinical necessity for the safe application of transpedicular screws in posterior spine instrumentation [8]. Furthermore, spine surgeons, in order to avoid serious intraoperative complications, have to assess carefully the morphometry of the vertebrae/pedicles preoperatively for patients undergoing transpedicular spine fixation. The aim of this study was to create a morphometric characteristic computed tomography (CT)-based database of thoracolumbar and lumbar (T9–L5) vertebrae for both sexes in the Greek population and to compare these findings with the analogous reported values in the available literature. Those individuals were picked from a pool of patients suffering vascular problems (mainly aortic aneurysmal disease) and had CT-angiography (mainly for abdominal aortic issues). We choose the T9 through L5 vertebrae in order to achieve homogeneity of the sample (visual vertebrae in all patients), since most of the CT scans did not involve the mid and upper thoracic vertebrae. To the authors’ knowledge, there is only one study in the literature regarding the pedicles’ dimensions in the Greek population [9].

## Material and methods

After obtaining the hospital’s IRB approval for this research study, a CT-based morphometric study from the ninth thoracic vertebra (T9) to the fifth lumbar vertebra (L5) for both sexes in the Greek population was carried out.

### The population studied

The CT scan images from T9 to L5 vertebrae were studied in 100 adults, age from 33 to 87 years old (mean 70 ± 8.73 years). There were 79 males and 21 females who were scheduled for vascular operations. Participants with a history of spinal surgery, vertebrae fractures, deformities, osteoporosis, and pre-existing spinal pathology were excluded from the study. Several dimensions of the vertebral bodies and the pedicles were measured.

### The measurements

**Fig. 1:**
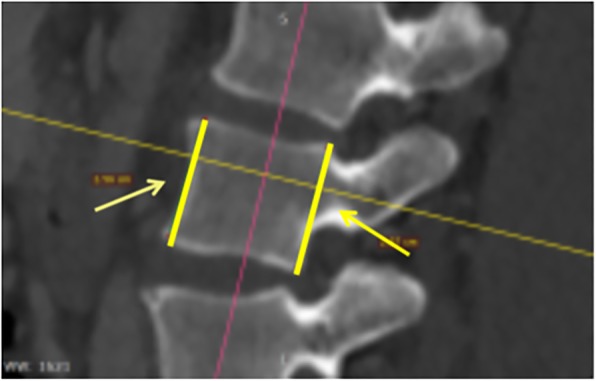
The anterior vertebral body height (AVBH) and posterior vertebral body height (PVBH)

**Fig. 2:**
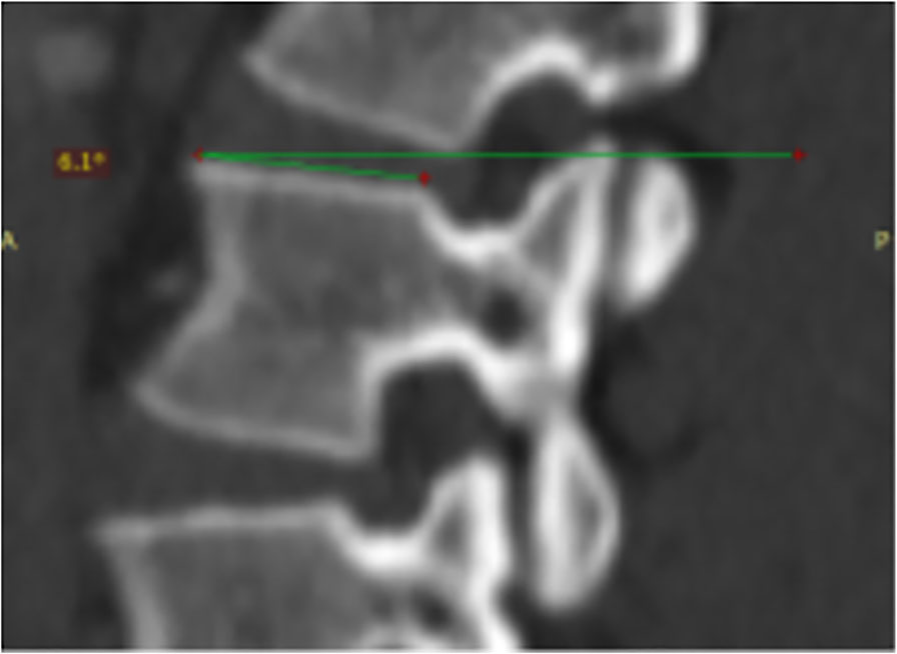
The angle formed by the upper end plate of vertebral body with the horizontal line in the sagittal plane

**Fig. 3:**
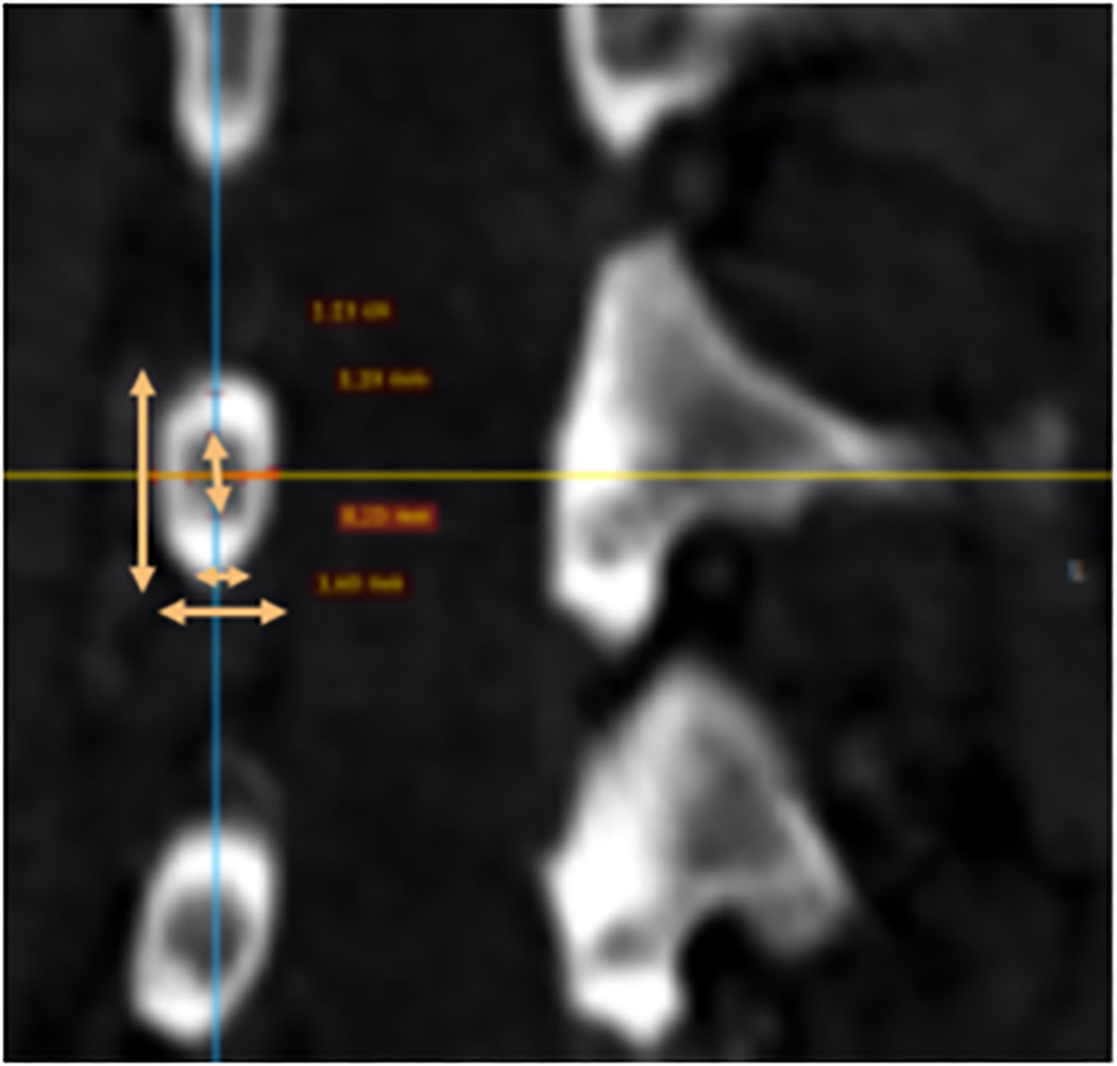
The inner cancellous and outer cortical pedicle height and width

**Fig. 4:**
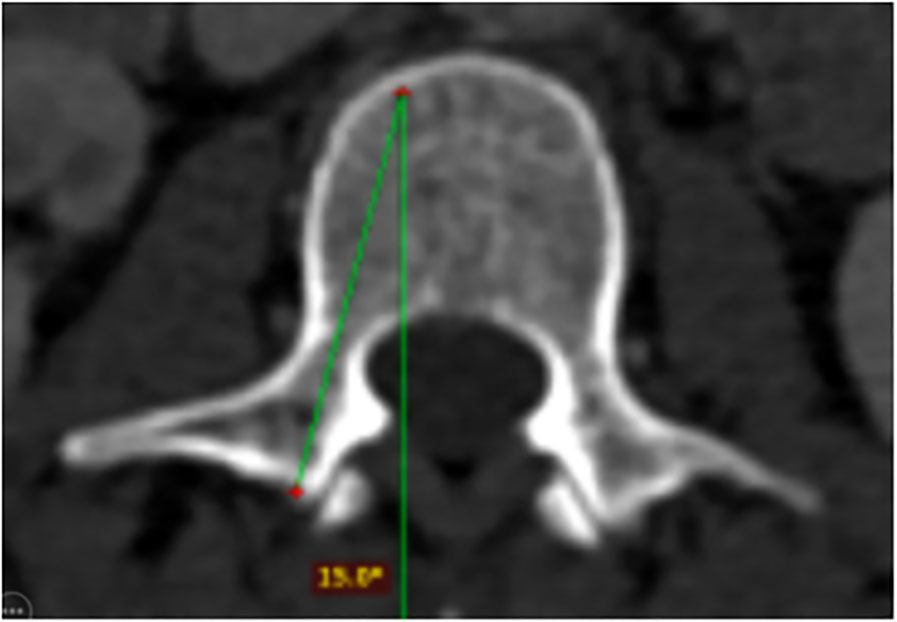
The angle formed by the longitudinal trajectory of the right- and left-sided pedicles, and the midline anteroposterior axis of the vertebra (PAA)

**Fig. 5:**
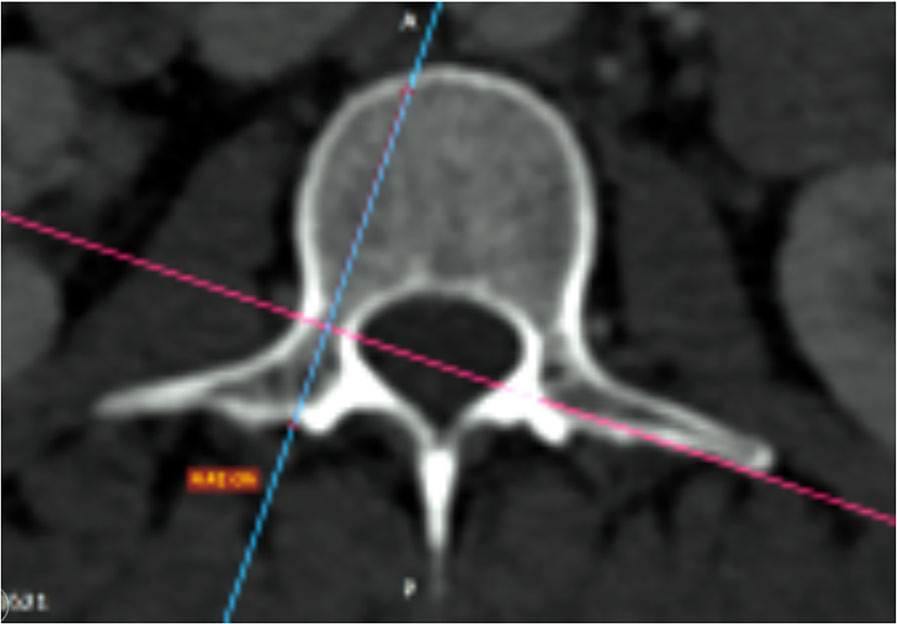
The postero-anterior trajectory’s length of the pedicle from the entry point of a hypothetical screw to the anterior cortex of the vertebra (PTLP) for the right- and left-sided pedicles

The anterior vertebral body height (AVBH), the posterior vertebral body height (PVBH), (Fig. 1), the angle of the upper end plate of vertebral body with the horizontal line in the sagittal plane (Fig. 2), the inner cancellous and outer cortical pedicle height and width (Fig. 3), the angle formed by the longitudinal trajectory of the right- and left-sided pedicles and the midline anteroposterior axis of the vertebra (pedicle axis angle (PAA)) (Fig. 4), and the postero-anterior trajectory’s length of the pedicle from the hypothetical entry point of the screw to the anterior cortex of the vertebra (PTLP) for the right- and left-sided pedicles (Fig. 5) for each sex were calculated.

### Statistical analysis

The collected data were statistically analyzed using the SAS/STAT software 3.1.3 and SPSS version 22. The non-parametric statistical technique of Mann-Whitney *U* test was used to compare the same parameters in males and females. Standard deviations (SD) and *p* values of all vertebral level were calculated separately. The statistical significance was set at the level of *p* < 0.05. The reliability study was also implemented.

## Results

The maximum AVBH for both sexes was found at the L5 vertebra with a mean of 28.47 mm (SD ± 2.55 mm) for males and 26.48 mm (SD ± 1.61 mm) for females, respectively. The maximum PVBH in males was at L1 vertebra with a mean of 27.77 mm (SD ± 1.64 mm), while in females at L2 vertebra with a mean of 27.11 mm (SD ± 1.27 mm). The minimum AVBH and PVBH, for both sexes, were at T9 with a mean of 20.29 mm (SD ± 1.79 mm) and 22.14 mm (SD ± 1.38 mm) for males and 18.83 mm (SD ± 1.36 mm) and 20.37 mm (SD ± 0.98 mm) for females, respectively. The mean AVBH increased gradually from T9 to L5, while the mean PVBH gradually increased from T9 to the L1 in males and L2 in females and then it decreased to the L5. The widest angle formed by the end plate of the vertebral body with the horizontal line in the sagittal plane, for males and females, was at L1 vertebra with a mean of 7.6° (SD ± 2.18°) in males and 9.5° (SD ± 6.56°) in females, respectively. The narrowest angle for males and females was found at L5 vertebra with a mean of − 15.79° (SD ± 478°) for males and − 16.32° (SD ± 9.66°) for females. The angle of the end plate of the vertebral body with the horizontal line in the sagittal plane increased from T9 to L1 vertebra and from L1 it decreased to L5 vertebra, with a negative angle meaning that the vertebra angled caudally.

Regarding the left pedicle, the largest pedicle height, for both sexes, was found at T11 with a mean inner cancellous height 12.86 mm (SD ± 1.26 mm) and the widest outer cortical 18.82 mm (SD ± 1.37 mm) for males and 10.24 mm (SD ± 1.88 mm) and 16.19 mm (SD ± 2.1 mm) for females. The smallest pedicle height for males was found at L5 with the inner cancellous and outer cortical height 7.24 mm (SD ± 1.39 mm) and 11.96 mm (SD ± 1.8 mm), respectively. For females, the smallest pedicle height was at T9, with a mean of 7.59 mm (SD ± 0.83 mm) and 12.39 mm (SD ± 0.82 mm) for the inner cancellous and outer cortical height, respectively. The mean pedicle height increased gradually from T9 to T11 and from T11 it decreased to L5. The widest pedicle width, for both sexes was at L5 vertebra with the mean inner cancellous and outer cortical width 11.57 mm (SD ± 1.97 mm) and 17.08 mm (SD ± 1.97 mm) for males and 12.7 mm (SD ± 4.25 mm) and 16.27 mm (SD ± 3.27 mm) for females, respectively. The narrowest pedicle width for males and females was found at T9 with a mean of 3.09 mm (SD ± 1.06 mm) for inner cancellous and 6.11 mm (SD ± 1.47 mm) for outer cortical for males, and 3.35 mm (SD ± 0.99 mm) and 6.8 mm (SD ± 1.35 mm) for female participants, respectively. The mean width of the left pedicle increased from T9 to L5 vertebra, with the narrowest inner cancellous at T9 and T10 vertebrae. The widest PAA was found at the L5 vertebra with a mean angle of 26.23° (SD ± 2.65°) for males and 23.63° (SD ± 4.59°) for females. The narrowest mean PAA in males was at T12 vertebra with a mean of 9.4° (SD ± 3.86°), while in females was at T9 with a mean of 9° (SD ± 3.67°). The mean PAA increased from thoracic vertebrae to L5. The longest PTLP was at the level of L4 with a mean of 55.31 mm (SD ± 4.52 mm) for males and 48.7 mm (SD ± 4.17 mm) for females. The shortest PTLP in males was at T9 with a mean of 47.2 mm (SD ± 3.18 mm) and in females at T12 with a mean of 40.95 mm (SD ± 3.79 mm). The mean PTLP increased from the thoracic vertebrae to L5, with the shortest at T9 vertebra.

Regarding the right pedicle, the longest height was at T12 with the mean inner cancellous and outer cortical height of 13.03 mm (SD ± 2.01 mm) and 18.01 mm (SD ± 1.56 mm) for males and 10.24 mm (SD ± 1.23 mm) and 16.14 mm (SD ± 1.37 mm) for females. The shortest pedicle height was found at L5 vertebra, with the mean inner cancellous of 6.61 mm (SD ± 1.16 mm) and the outer cortical of 11, 68 mm (SD ± 0.92 mm) in males and 7.76 mm (SD ± 1.19 mm) and 12.59 mm (SD ± 2.5 mm) in females, respectively. The mean height of the right pedicle increased from T9 to T12 and from L1 decreased to L5. The widest pedicle width was found at L5, with the mean inner cancellous and outer cortical width of 11.3 mm (SD ± 2.86 mm) and 16.34 mm (SD ± 2.98 mm) for males and 12 mm (SD ± 3.18 mm) and 15.69 mm (SD ± 2.59 mm) for females, respectively. The narrowest pedicle width was at T9 with a mean of 2.83 mm (SD ± 0.97 mm) and 5.68 mm (SD ± 1.35 mm) for inner and outer width for males and 3.2 mm (SD ± 0.97 mm) and 6.4 mm (SD ± 1.42 mm) for females. The mean width of the right pedicle increased from T9 to T12 regarding the thoracic vertebrae and from L1 to L5 regarding the lumbar vertebrae, with the narrowest inner cancellous width at T9 and T10. The greatest PAA was at L5 with a mean of 25.7° (SD ± 5.19°) in males and 25.56° (SD ± 5.31°) in females. The smallest PAA in males was found at the level of T9 with a mean of 9.51° (SD ± 1.74°); however, in females the T10 vertebra had the smallest mean PAA with a mean of 8.86° (SD ± 8.13°). The longest PTLP was at the level of L3 with a mean of 54.86 mm (SD ± 3.18 mm) for males and 49.01 mm (SD ± 2.97 mm) for females. The shortest PTLP was at T9 with a mean of 48.68 mm (SD ± 3.58 mm) for males and 39.44 (SD ± 3 mm) for females. The mean PTLP of the right pedicle increased from T9 to L4 in males and from T9 to L3 in females, with the shortest at T9 vertebra.

Regarding the statistical difference between sexes for each level of the thoracolumbar and lumbar spine from T9 to L5, it was found that in all vertebrae, a statistically significant difference (*p* < 0.0001) was observed for the PLTP of the left- and right-sided pedicles. Furthermore, at the L5 level, a statistically significant difference was observed for PVBH (*p* < 0.0091) and inner cancellous height of the left pedicle (*p* < 0.0193). At L4 level for inner cancellous and outer cortical width of both pedicles (left pedicle *p* < 0.0028 and *p* < 0.0047, and right pedicle *p* < 0.0042 and 0.014, respectively). At L3 level statistically significant difference was observed for PVBH (*p* < 0.0462) for inner and outer height and width of left pedicle, (*p* < 0.0002, *p* < 0.0193, *p* < 0.0039, *p* < 0.0022, respectively) and also inner and outer height of the right pedicle (*p* < 0.0045, *p* < 0.0003) and only at the inner width (*p* < 0.025) but not at the outer one. At L2 level for the angle of the upper end plate of vertebral body with horizontal line in the sagittal plane (*p* < 0.0038), for the inner height (*p* < 0.0048), and the inner and outer width of the left pedicle (*p* < 0.0006 and *p* < 0.0497), respectively.

**Fig. 6:**
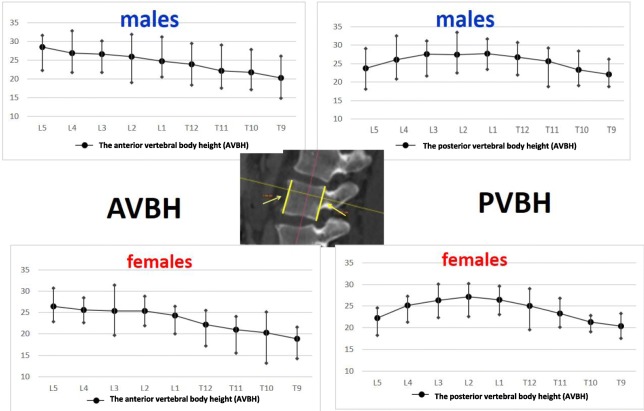
The anterior vertebral body height (AVBH) in millimeters and posterior vertebral body height (PVBH) in millimeters, and the ±SD from T9 to L5 for males and females

**Fig. 7:**
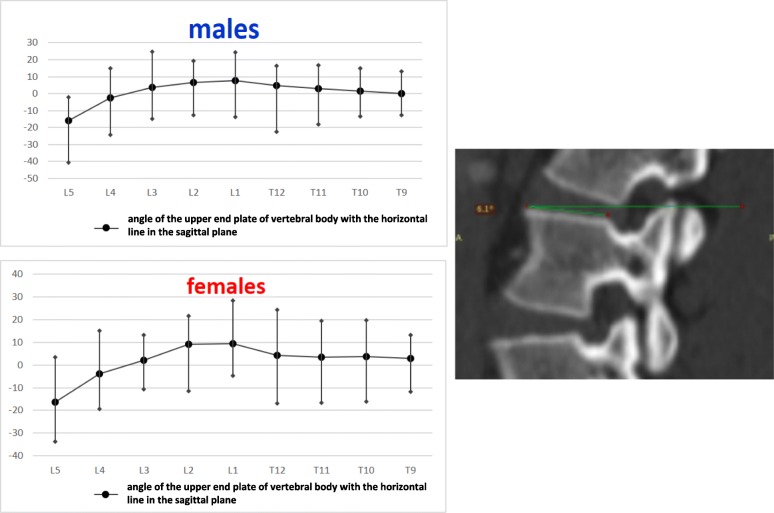
The angle formed by the upper end plate of vertebral body and the horizontal line in the sagittal plane in degrees and the ±SD from T9 to L5 for males and females. It is deemed positive (+) if the angle opens posteriorly above the horizontal and negative (−) if the angle opens anteriorly below the horizontal

**Fig. 8:**
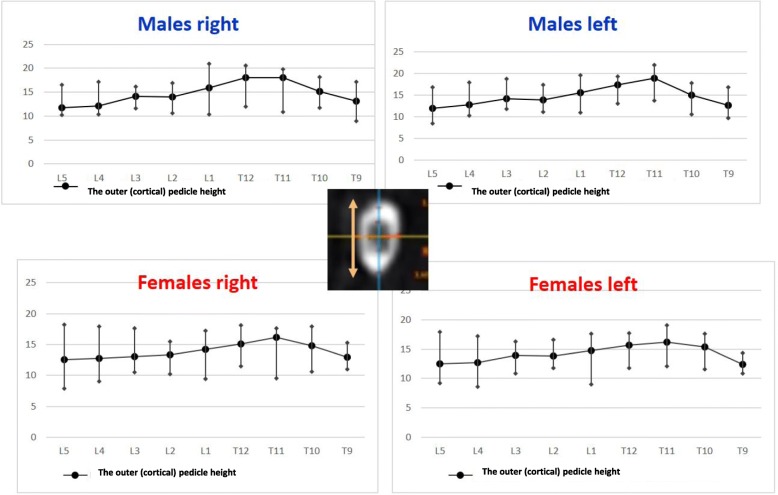
The outer cortical right and left pedicle height and the ±SD in millimeters from T9 to L5 for males and females

**Fig. 9:**
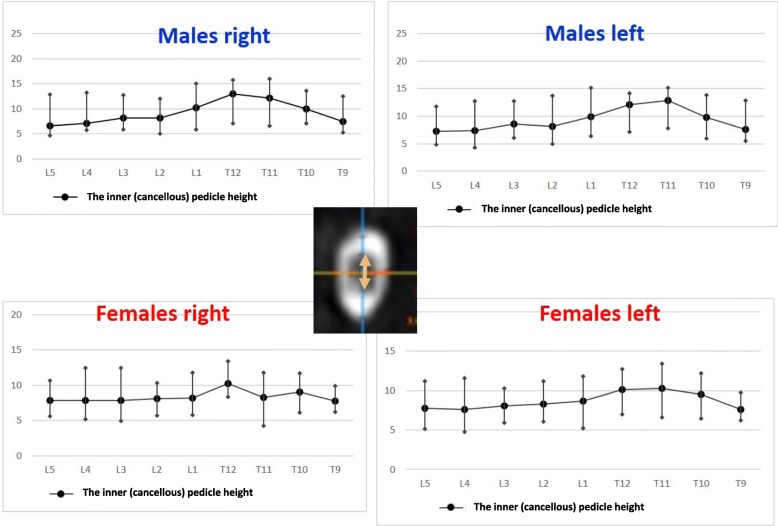
The inner cancellous right and left pedicle height and the ±SD in millimeters from T9 to L5 for males and females

**Fig. 10:**
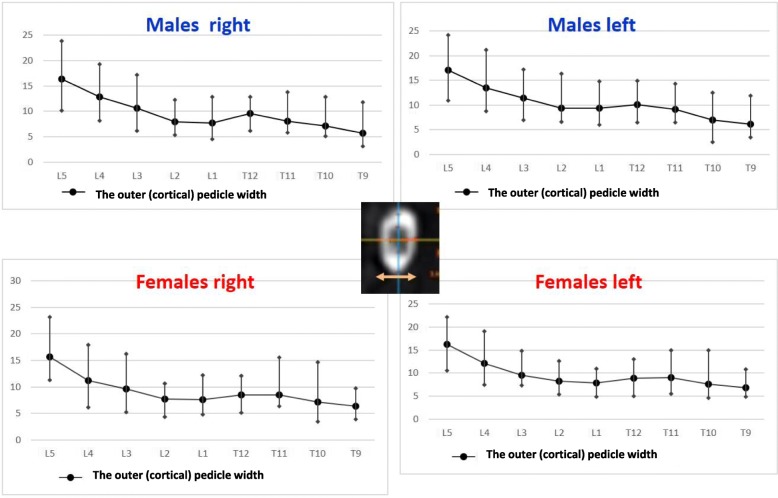
The outer cortical right and left pedicle width and the ±SD in millimeters from T9 to L5 for males and females

**Fig. 11:**
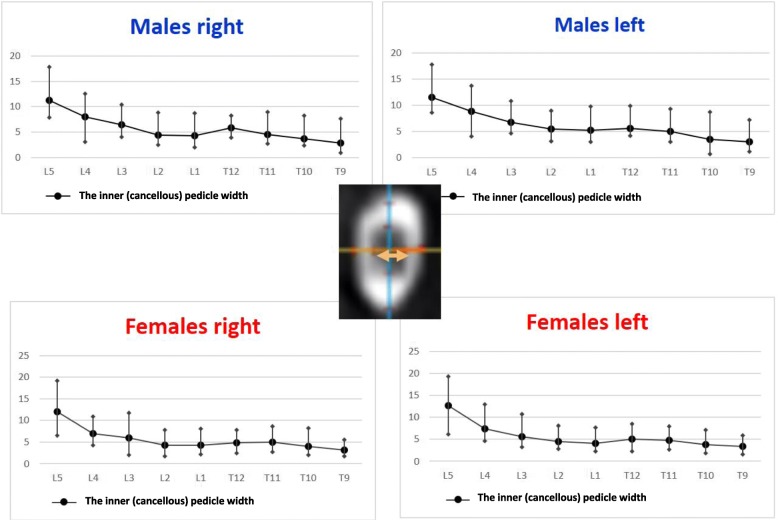
The inner cancellous right and left pedicle width and the ±SD in millimeters from T9 to L5 for males and females

**Fig. 12:**
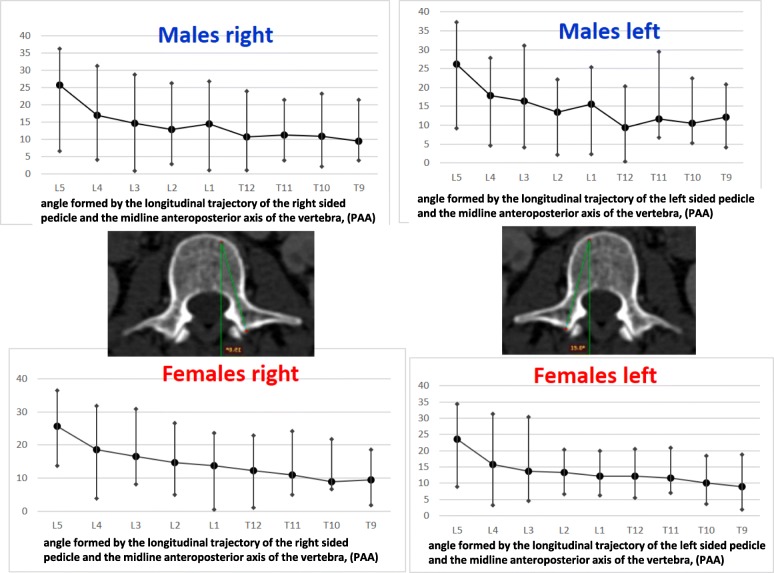
The angle PAA in degrees and the ±SD in degrees from T9 to L5 for males and females

**Fig. 13:**
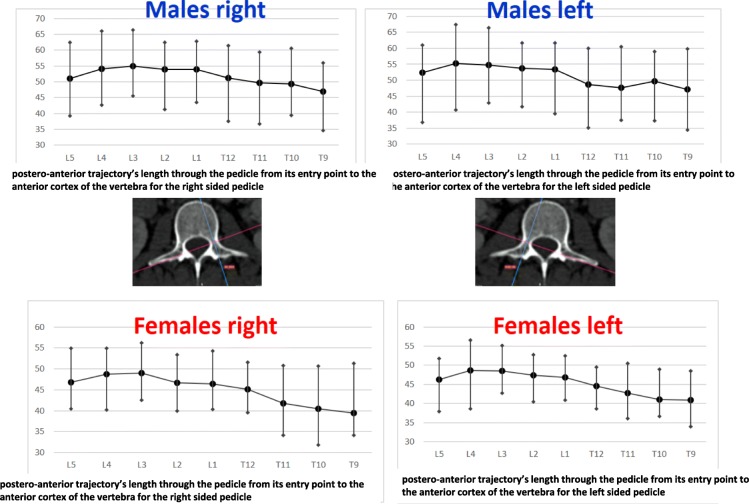
The PTLP for the right- and left-sided pedicle and the ±SD in millimeters from T9 to L5 for males and females

The right pedicle at L1, there was a statistically significant difference in all the parameters except the angle of the upper end plate of vertebral body with horizontal line in sagittal plane, the left outer height and width, the right outer height, and the right PAA. At T12 level, a statistically significant difference (*p* < 0.0001) was observed for left and right pedicle inner height. At T11 level for left and right pedicle inner height and for the PVBH. At T10 level, a statistically significant difference (*p* < 0.0001) was observed for PVBH. As for the Τ9 level, the difference (*p* < 0.0001) was for the PVBH. The results are also presented in Figs. 6, 7, 8, 9, 10, 11, 12, and 13.

### The reliability study

In the CTs, the slice thickness was ranging from 5 mm to 0.5 mm. For the intra-class, correlation coefficient with 95% confidence interval was 0.998 for the length measurements, and the intra-class correlation coefficient with 95% confidence interval was 0.971 for the angular measurements. The reliability results were better when the slice thickness was smaller. The two-way mixed model for ICC was used (using the IBM SPSS version 22).

## Discussion

The pedicle screws are used for the posterior spine fixation to treat various spine disorders or trauma. However, inaccurate placement is relatively common even when placement is performed under fluoroscopic control [5], and it can cause severe vascular and neurological complications [10]. However, in clinical practice, for safe and accurate screw placement, the screw dimensions as well as the proper position of the screw are essential. Optimum diameter of the pedicle screw is necessary to maximize the rigidity of the construct, as larger diameter screws may break the pedicle, while screws with narrower diameter do not offer sufficient resistance to pull out [11]. Regarding the screw length, short screws reduce the rigidity of the construct, while long screws may penetrate the anterior wall of the vertebral body and injure vital structures. Biomechanical studies have shown that the pull-out strength of a pedicle screw at 85% insertion depth is similar to the pullout strength at 100% insertion depth [12]. Another important parameter for proper screw position is the knowledge of PAA for each vertebra, in order to prevent medial or lateral pedicle wall breach [13]. Therefore, a profound knowledge of the morphometric characteristics of the vertebrae is needed [14].

Some morphometric measurements of the vertebrae and pedicles significantly differ among different ethnic groups and preoperative software-based morphometric data should be collected for preoperative planning [9, 15–19]. Vertebral morphometric measurements of the Greek population shared some similarities and differences with other ethnic groups. There is only one study in the literature regarding the pedicle dimensions in the Greek population. Christodoulou et al. [9] studied 12 human cadaveric spines (5 women and 7 men) with a mean age of 69.6 years (range 62 to 84 years) at the time of death. The authors measured the transverse and sagittal outside pedicle isthmus widths, the internal transverse diameter, and cortex width of pedicles with electronic calipers. In our study, the widest outer cortical pedicle width was at L5 with a mean of 17.08 mm (SD ± 1.97 mm) at left pedicle in males. This is in accordance with the results of Christodoulou et al. [9] who found that the widest transverse diameter was at the same level of L5 with a mean of 11.3 mm (range 7.55–15.46 mm). Zindrick et al. [20], who conducted one of the largest morphometric measurements of the pedicles by CT in Western populations, found the same level with a mean of 18.0 mm (range 9.1–29.0 mm), similar to our measurements. Regarding the widest inner cancellous pedicle width, in this study, it was at the L5 vertebra with a mean of 11.3 mm (SD ± 2.68 mm) at the right pedicle in males. However, Christodoulou et al. [9] found the widest inner at L4 level with a mean of 8.26 mm (range 7.10–9.23 mm). In this report, as far as the largest outer pedicle height is concern, it was found at T11 left pedicle in males with a mean of 18.82 (SD ± 1.37 mm). This finding is also in accordance with the results of Christodoulou et al. [9] who found the largest height at the same level with a mean of 17.23 mm (range 14.84–19.57 mm) and also with the results of Zindrick et al. [20] with a mean value of 17.4 mm (range 12.5–24.1 mm). The narrowest inner pedicle width was observed at T9 and T10 vertebrae in our study, with a mean from 2.83 mm (SD ± 0.97 mm) to 4.00 mm (SD ± 1.43 mm) for male and female population, respectively. However, Christodoulou et al. [9] stated that a 5-mm-diameter screw may safely be inserted at the levels of T9 vertebra. Zindrick et al. [20] also found that the mean inner pedicle width at T9 and T10 was larger with 6.1 mm (range 3.7–9.0 mm) at T9 and 6.3 mm (range 3.1–8.5 mm) at T10 vertebra. In clinical practice, the diameter of pedicle screws for thoracolumbar levels range from 4 mm to 7 mm, with 1.0 mm clearance. [12]. Based on the analysis of the present study, in the Greek population, the pedicles of T9 and T10 vertebrae may hardly accommodate a 4.0-mm pedicle screw, due to the narrow inner cancellous pedicle width. Studies have shown that pedicles between T4 and T8 should be measured on CT scans before an operation, because they might not be suitable for fixation with screw due to their narrow width [14, 16]. Based on the results of this study, T9 and T10 must also be included in the CT scan preoperative, especially for the Greek population. Regarding the PAA of the pedicles in this study, the largest PAA was at L5 with a mean of 26.23° (SD ± 2.65°), at the left pedicle in males, while the smallest was found at T9 with a mean of 9° (SD ± 3.67°), at the left pedicle in females. Zindrick et al. [20] also stated that the largest angle was seen at L5 with the mean pedicle of 29.8° (range 19.0–44.0°); however, the shallowest was at T1, with the mean PAA at T9 vertebra 7.6° (range 0.0–10.5°). The longest PTLP in our study was 55.31 mm (SD ± 4.52 mm) at L4, at the left pedicle in males and the shortest at T9, at the right pedicle in females, with a mean of 39.44 mm (SD ± 3 mm). However, Zindrick et al. [20] found the longest PTLP (through pedicle axis) at L2 and L3 that was 51.9 mm (ranges 45.0–58.0 and 42.0–62.0 mm, respectively), Olsewski et al. [21] at L4, and Vaccaro et al. [22] at T12.

As far as the differences between both sexes are concerned, Christodoulou et al. [9] concluded that regarding the internal diameter in the lumbar spine, there was a difference between males and females especially at L3 levels (*p* < 0.05), and almost in all levels regarding the cortex width. In our report, the more pronounced statistically significant difference (*p* < 0.0001) between the two sexes was the PTLP at all vertebral levels from T9 to L5. In this study, the L5 vertebra was found to have the largest AVBH, PAA, and the width of pedicles in both sexes. AVBH was found to be smaller compared to the PVBH at T9–L3 vertebral bodies, almost equal at L4 level and greater only at L5 vertebral. This observation may be due to the normal physiological lordosis present in the thoracolumbar region.

Although this study provides important information about the morphometry of vertebrae in the Greek population, it has some limitations. The current findings were obtained from patients visiting a single hospital, and possible differences in the morphometric parameters might exist between Greek people from different geographic regions (i.e., South, West, Central Greece). The male patients were almost four times more than the female ones (79 men and 21 women). Another limitation was the variety of slice thickness at the CT scans (range from 5 mm to 0.5 mm) and at the axial CT scan, which provides only a two-dimensional view of the three-dimensional shape of the pedicles. It is however recommended that the preoperative CT imaging be implemented with thinner slices as possible for a more accurate assessment of the morphometric characteristics of the vertebrae.

## Conclusions

In conclusion, this study provides a database of morphometric characteristics on thoracolumbar and lumbar vertebrae from T9 to L5 in the Greek population. This database may prove to be of great significance for forthcoming comparative studies. Future studies would also establish the morphometric characteristics of the thoracic regions in the Greek population. It can also serve as a basis in order to detect pathological changes in the spine and furthermore to plan operative interventions.

## Abbreviations

AVBH: Anterior vertebral body height
CT: Computed tomography
L: Lumbar
PAA: Pedicle axis angle
PVBH: Posterior vertebral body height
T: Thoracic
